# Automation of Healthcare-Associated Infections (HAIs) Areas for Opportunity Through the Use of Technology

**DOI:** 10.1017/ash.2021.6

**Published:** 2021-07-29

**Authors:** Meri Pearson

## Abstract

**Background:** A large healthcare system in Georgia implemented an enhanced electronic infection surveillance system that is incorporated into the electronic medical record (EMR). Prior to the implementation of this electronic infection surveillance system, the infection prevention (IP) team performed healthcare-associated infection (HAI) surveillance through a locally created system that did not interface with their EMR. Each HAI identified undergoes a robust analysis for opportunities depending on the infection type by manual abstraction from the EMR, which is stored in a spreadsheet. One disadvantage of this spreadsheet is that only 1 person can enter data at a time. The coronavirus disease 2019 (COVID-19) pandemic has presented many challenges for healthcare facilities including shifting resources from HAI prevention programs. These programs include the investigations performed to identify risk factors that can aid in preventing future infections. Due to the necessity to increase efficiency in the current pandemic, the IP team proposed using technology to automate our HAI investigation process. **Method:** The IP team and the business intelligence (BI) team met to determine whether data completed in the electronic infection surveillance system could flow into an interactive data visualization software that is currently used for other HAI prevention dashboards. The existing spreadsheet was reviewed to select variables essential for HAI investigations and for which the data existed in the EMR. The BI team worked to find the correct data tables within the EMR so that the data could automatically refresh daily in the data visualization software. **Result:** The BI team was able to correctly identify variables used in the previously manual HAI investigation process within the EMR to interface with the data visualization software. This automation of investigations allows quick analysis of trends and areas of opportunity to prevent future HAIs. **Conclusion:** This utilization of technology can be applied to other healthcare facilities with similar software systems to streamline IP workflows. The automation of quickly and efficiently recognizing areas of opportunity allows IPs more time to facilitate the prevention of HAIs in other ways.

**Funding:** No

**Disclosures:** None

